# Convolutional Neural Networks for Segmenting Cerebellar Fissures from Magnetic Resonance Imaging

**DOI:** 10.3390/s22041345

**Published:** 2022-02-10

**Authors:** Robin Cabeza-Ruiz, Luis Velázquez-Pérez, Alejandro Linares-Barranco, Roberto Pérez-Rodríguez

**Affiliations:** 1 CAD/CAM Study Centre, University of Holguín, Holguín 80100, Cuba; roberto.perez@uho.edu.cu; 2 Cuban Academy of Sciences, Havana 10200, Cuba; velazq63@gmail.com; 3 Centre for the Research and Rehabilitation of Hereditary Ataxias, Holguín 80100, Cuba; 4 Robotics and Tech. of Computers Lab, University of Seville, 41012 Seville, Spain; alinares@us.es; 5 Escuela Politécnica Superior (EPS), University of Seville, 41011 Seville, Spain; 6 Smart Computer Systems Research and Engineering Lab (SCORE), Research Institute of Computer Engineering (I3US), University of Seville, 41012 Seville, Spain

**Keywords:** convolutional neural network, cerebellum segmentation, neurodegenerative disease, cerebellar fissures, magnetic resonance imaging

## Abstract

The human cerebellum plays an important role in coordination tasks. Diseases such as spinocerebellar ataxias tend to cause severe damage to the cerebellum, leading patients to a progressive loss of motor coordination. The detection of such damages can help specialists to approximate the state of the disease, as well as to perform statistical analysis, in order to propose treatment therapies for the patients. Manual segmentation of such patterns from magnetic resonance imaging is a very difficult and time-consuming task, and is not a viable solution if the number of images to process is relatively large. In recent years, deep learning techniques such as convolutional neural networks (CNNs or convnets) have experienced an increased development, and many researchers have used them to automatically segment medical images. In this research, we propose the use of convolutional neural networks for automatically segmenting the cerebellar fissures from brain magnetic resonance imaging. Three models are presented, based on the same CNN architecture, for obtaining three different binary masks: fissures, cerebellum with fissures, and cerebellum without fissures. The models perform well in terms of precision and efficiency. Evaluation results show that convnets can be trained for such purposes, and could be considered as additional tools in the diagnosis and characterization of neurodegenerative diseases.

## 1. Introduction

The human cerebellum plays an essential role in critical tasks, like motor coordination and cognition, and is related to other functions, e.g., language and emotions [1,2]. Diseases like spinocerebellar ataxias (SCAs), multiple sclerosis (MD), or Alzheimer’s disease (AD), are known to cause damage in the cerebellum, conducting patients to progressive loss in such functions and, in some cases, to premature death [3]. Cerebellar damage caused by such diseases occurs in the form of degeneration, reducing the cerebellar volume. The damage can be seen as large fissures, and grows with the progression of the disease. Knowing how to observe such fissures allows specialists to obtain some important characteristics from the patients, like volume loss related to the specific disease.

Segmentation of magnetic resonance imaging (MRI) is often performed, and clinicians make research with several patients, with the goal of learning more about the disease, and how to treat it better. However, manual segmentation of MRIs is a complex and time-consuming task, and becomes impractical as the number of images increases. For that reason, computational tools are required for performing those processes automatically.

Automated cerebellum processing from MRIs has been addressed by several authors, in studies mainly oriented to the delineation and volume calculation of the whole organ and its lobules [1,4,5,6,7,8], deep nuclei segmentation [9], and gray/white matter segmentation [10]. Diedrichsen et al. [7] proposed a probabilistic atlas of the human cerebellum, and performed automatic cerebellum parcellation by combining it with registered images. Weier et al. [8] parcellated cerebellum using patch-based label-fusion and a template library composed of manually labelled images. Romero et al. [5] proposed CERES, which is currently one state-of-the-art pipeline for cerebellar segmentation and parcellation, based on atlas templates and several registration steps for each image to be processed. Manjón and Coupé [10] proposed VolBrain as a tool for subcortical structure segmentation, based on multi-atlas label-fusion. Dolz, Desrosiers and Ben Ayed [11] used a fully convolutional neural network which has been tested in [6] for cerebellar parcellation, obtaining good results. Han et al. [1] proposed the ACAPULCO pipeline, which relies on convolutional neural networks, for performing cerebellar parcellation from MRIs. Kim et al. [9] performed deep cerebellar nuclei segmentation using a fully connected densenet. Thyreau and Taki [12] used convolutional neural networks for brain cortical tissue parcellation from an initial brain mask.

Currently, two of the top-most ranked applications on cerebellar segmentation and parcellation are CERES and ACAPULCO. CERES is based on multi-atlas segmentation, and consists of a pipeline which includes several registration stages, inhomogeneity corrections, and intensity normalizations. It has outperformed all other solutions in the study made by Carass et al. [6]. ACAPULCO is based on convolutional neural networks. The system uses a first CNN to find a bounding box of the cerebellum, and a second, deeper CNN to divide the organ into 28 regions. As reported by Han et al. [1] it surpassed an improved version of CERES in the segmentation of various cerebellar lobules.

In the last decade, convolutional neural networks [13] have experimented a rapid development, as the number of researchers using them for medical image processing grows, in systems where performance is an important factor [14,15,16,17]. Specifically, for brain MRI processing, convnets have been successfully applied in segmentation and classification tasks, predicting the stage of Alzheimer’s disease [18], cerebellum [4] and brain parcellation [19], and tumor detection and segmentation [20].

**Figure 1:**
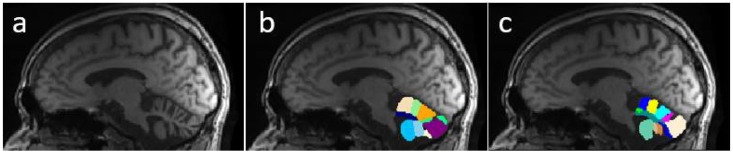
Comparison between segmentations on MRI of SCA2 patient. In (**a**) the original imaging, in (**b**) segmentation produced by ACAPULCO, and in (**c**) segmentation obtained by CERES.

Despite the excellence of the existing methods and the reported results, none of this research is oriented to correctly segment and determine all important fissures in cerebellum of patients with neurodegenerative diseases. Figure 1 shows a comparison between segmentations produced by CERES and ACAPULCO for one magnetic resonance from a SCA2 patient with severe cerebellar atrophy. It can be seen that CERES made a better recognition of increased fissures than ACAPULCO, however, some of them have been incorrectly classified as cerebellar tissue. This phenomenon must be related to the training images and labels for both methods, but it should have great impact on the calculation of volumes for the affected parts. As the fissures are classified as cerebellar tissue, the resulting volumes should be larger than the actual ones, giving an incorrect idea of the atrophy produced in the patient’s cerebellum. Images were generated with ITK-Snap software [21], CERES segmentation was obtained through the web portal (https://www.volbrain.upv.es/, accessed on 5 December 2021), and ACAPULCO segmentation was obtained by using a docker container shared by the authors in the original paper [1].

This article proposes the use of convolutional neural networks for segmenting the cerebellum and its fissures. The study comprises analysis over three CNN models, based in the same architecture, for obtaining binary masks of the whole cerebellum without fissures, the cerebellum with its fissures, and the fissures mask itself. Our analysis demonstrates the feasibility of convnets for such tasks. We think that the existence of tools for recognizing the cerebellar fissures from brain MRIs of patients with cerebellar disorders should improve the automated volume estimation currently applied by the aforementioned research, bringing the calculations closer to the real values. Produced segmentations might give an idea of the total volume loss in patients, as well as the stage and progression of the disease itself. As part of the performed analysis, our system is compared with ACAPULCO and CERES, demonstrating an improvement in the segmentation of cerebellar tissue with a correct estimate of the fissures. Additionally, a simple procedure is proposed to help in the construction of similar datasets, relying on an existing mask of the structure to be segmented.

## 2. Materials and Methods

### 2.1. Models and Implementation Details

**Figure 2:**
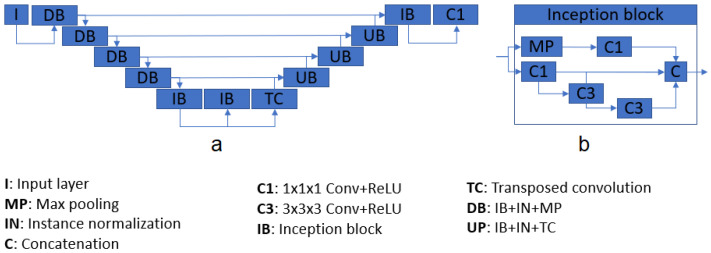
Architecture diagram (**a**), and inception module pipeline (**b**).

Our three models are built upon the same U-Net architecture. The only differences between models are the labels used for training. Table 1 shows the difference between the three models. The proposed structure is based on U-Net [22], a well-known CNN architecture which takes advantage of feature maps created in previous steps. This characteristic gives the network the ability of processing more complex images while reducing the computational requirements. The system consists of four down- and up-sample steps, composed of inception modules [23] and instance normalization layers, and two chained inception modules as a bottleneck. Each inception module is composed of four convolutional layers, one max pooling operation, and a final concatenation. After each inception module, an instance normalization [1,24] layer processes the produced features. All the activation layers (one per convolution) are Rectified Linear Units (ReLU) [25]. Figure 2 shows the main architecture. The total number of inception modules used was 10, and the number of filters passed to them, in sequence, were 16, 16, 32, 64, and 128 for the contracting path. For the decoding section, the number of parameters were 128, 64, 32, 16, 16. Note that, for each inception module, the output size is four times the input size; e.g., a module with an input of size 128 will return an output with 512 feature maps. The final layer of the architecture consists of a convolutional layer with one single filter, returning the segmented mask from the input.

**Table 1:** Differences between the three used models.

Model Name	Desired Output	Reference Figure
M1	Binary mask with only cerebellar fissures	Figure 3g
M2	Binary mask of the cerebellum with its fissures	Figure 3h
M3	Binary mask of the cerebellum without fissures	Figure 3f

Implementation was made with Keras [26] and TensorFlow backend [27], using the Python 3.7 programming language, and the training was done on a 16 GB Tesla P100-SXM2 GPU, available through a Jupyter notebook on Google Colab (https://colab.research.google.com/, accessed on 5 December 2021). The used optimizer was Adam [28], with its default values. To avoid overfitting, a dropout of 0.3 was established after the last convolutional layer of each model. Rather than preparing a single model for predicting the three desired features, we trained separated ones for simplicity, making our task a single label segmentation problem. Finally, image cropping was done for reducing computational cost of algorithms. All images were cropped to a volume containing only the cerebellum.

### 2.2. Data Preparation and Dataset Construction

The used dataset consists of 24 magnetic resonances retrieved from the Cuban Neurosciences Center. The images belong to 15 patients, divided into three categories: five healthy controls, five presymptomatic carriers, and five patients diagnosed with spinocerebellar ataxia type 2 (SCA2). Presymptomatic carriers in this research are treated as patients, as it is well known that cerebellar atrophy due to SCA2 may be present long before the disease onset [29,30,31].

**Figure 3:**
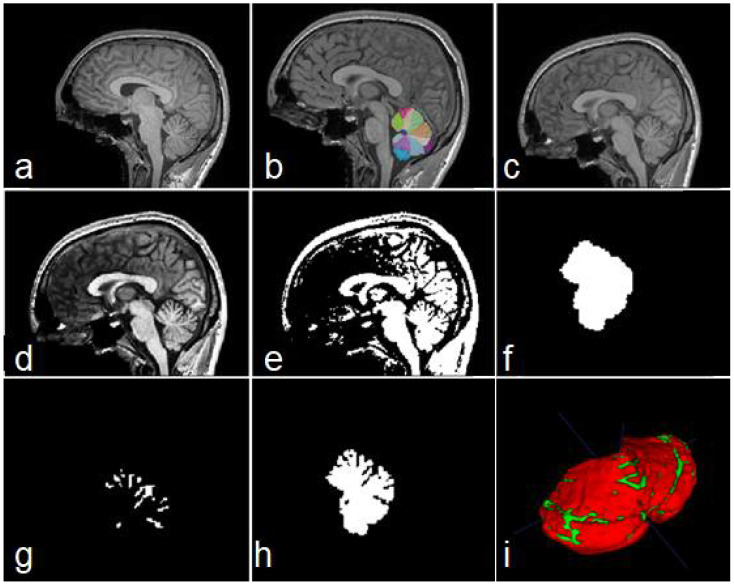
Steps of data construction procedure. Sagittal views of the original image (**a**), cerebellar mask obtained with ACAPULCO (**b**), result of BFC and registration (**c**), contrast-enhanced image (**d**), binary image obtained (**e**), feature map containing the whole cerebellar tissue (**f**), obtained fissures mask (**g**), and cerebellum with fissures (**h**). In (**i**) a 3D view of the union of (**g**,**h**); red color represents the cerebellar tissue, and green color shows the fissures.

Building a manually labelled dataset from 3D images is a very difficult task. For this reason, we created a simple procedure for the preparation of our dataset. For each MRI, the following steps were applied:

1. Obtain a cerebellar mask, using any existent technique. See Figure 3b.
2. Bias Field Correction (BFC) for reducing intensity inhomogeneities. The algorithm used in this research was the N4 method [32].
3. Image registration to the 1 mm isotropic ICBM 2009c template [33] in MNI space. See Figure 3c.
4. Obtain a contrast-enhanced image (Figure 3d).
5. Binarize equalized image using any existent technique (Figure 3e).
6. Build a mask containing the cerebellar segmentation obtained in step 1 (output 1). See Figure 3f.
7. Build a feature map containing cerebellar fissures, by applying binary xor operation to outputs from steps 5 and 6 (output 2, Figure 3g,i).
8. Build a feature map containing the cerebellar tissue, with all its fissures, by subtracting output 1 from output 2 (output 3, Figure 3h,i).
9. Imaging cropping for reducing computational cost.

For the original cerebellum mask, any available tool can be used, but we highly recommend using ACAPULCO [1] or CERES [5], which are state-of-the-art pipelines for cerebellum parcellation. For this study, ACAPULCO was used, accessed through a docker image shared by the authors in the original paper. The segmented masks have been manually corrected, in order to eliminate any errors than can occur. Manual correction was done with the software ITK-Snap [21].

N4 bias field correction and rigid registration were performed with the ANTS suit [34], available at http://stnava.github.io/ANTs/ (accessed on 5 December 2021).

Enhanced-contrast images were obtained by following a pipeline of intensity normalization (Equation (1)), rescaling to range [1; 255], and histogram equalization. This contrast-enhanced image will serve as the input for the three segmentation models.

(1) $$i=\frac{i-mean\left(i\right)}{std\left(i\right)}$$

To obtain the binary maps, we computed the Otsu threshold [35], and kept only those voxels with an intensity higher than the calculated threshold. If the original imaging contains a high contrast, some errors may be carried through this procedure, obtaining an incorrect binary map (i.e., several parts of the cerebellar tissue can be removed). For that reason, the binary images must be visually inspected and corrected.

For creating the mask parting from the original cerebellum segmentation, we used the Morphological Snakes algorithm [36]. We applied this step as it improves border smoothness, and may be used to regularize segmentations created/corrected by different raters. The original implementation can be found at https://github.com/pmneila/morphsnakes (last accessed on 20 November 2021).

Steps 6, 7 and 8 from the algorithm (outputs 1, 2 and 3), are used as the output maps for the system training. They correspond to the whole cerebellar mask, cerebellar fissures, and cerebellum tissue with its fissures, respectively. The last step is optional, but recommendable if low computational resources are available.

By following the procedure, the construction of an entire dataset may be significantly reduced, since user interaction is limited only to correcting errors, which in some cases are minimal.

### 2.3. Analysis Description

From the 24 images composing our dataset, 17 were used for training, two for validation, and five for testing purposes. To avoid overfitting, data augmentation was applied to those images on the train/validation partition. The images were augmented using combinations of rotations in the range [−10°, 10°] and shifts on random axes, in the range [–10, 10]. For every training/validation image, 40 new augmented images were created. The three models were trained during 120 epochs, and evaluations were made on the five unseen images.

For testing the robustness of trained models, we tested on subsets of other three datasets:

1. Ten magnetic resonances from the Hammers 2017 dataset (Hammers) [37]. The dataset contains 30 MRIs from healthy subjects, manually segmented by experts into 95 regions [38,39,40]. From the 95 labels, we used only 17 and 18, corresponding to left and right cerebellum, respectively.
2. Ten magnetic resonances from the Dallas Lifespan Brain Study dataset (DLBS) [41,42,43]. The dataset contains 315 MRIs of healthy people, some of them are healthy carriers of APOE gene. The initial cerebellar maps for this dataset were obtained by combining the labels from the output of ACAPULCO.
3. Seven magnetic resonances obtained from BrainWeb [44,45]. The site allows the construction of simulated MRIs from healthy people and MS patients, based on templates. The images used in this study were constructed simulating mild, moderate and severe MS lesions (http://www.bic.mni.mcgill.ca/brainweb/, last accessed on 5 December 2021).

As a preprocessing stage, steps 2–4 and 9 from the described procedure were applied. Therefore, our preprocess comprises bias field correction, registration to MNI space, contrast enhancement, and image cropping.

For the model predicting cerebellar fissures (M1), no postprocessing technique was applied. The evaluations were carried on the untouched outputs. In the case of the models responsible for segmenting cerebellum with and without fissures (M2 and M3, respectively), a selection of longest connected component was done, classifying only the biggest structure as cerebellar tissue.

Finally, for evaluating the impact of the current research, segmentations of model M2 were compared with the results of ACAPULCO and CERES.

### 2.4. Evaluation Metrics

Dice Score (DSC, F1-score), overlap coefficient (OC), specificity (SP, True negative rate, TNR), sensitivity (SN, True positive rate, TPR), and area under the ROI curve (AUC), are used as the evaluation metrics for the three models. *DSC* allows comparison of two volumes of the same dimensions through Equation (2) [46]:

(2) $$DSC=\frac{2\times \sum_{i}^{N}p_{i}g_{i}}{\sum_{i}^{N}p_{i}^{2}+\sum_{i}^{N}g_{i}^{2}}$$

where *N* represents the total number of voxels in one image, *p* belongs to the prediction volume, and *g* belongs to the ground truth volume. *SP* allows to quantify the proportion of those voxels that do not belong to the ground truth mask, and can be obtained with Equation (3) [47]:

(3) $$SP=\frac{TN}{FP+TN}$$

where *TP* and *TN* are the number of voxels which have been correctly recognized as part of the mask and part of the background, respectively, and *FP*, *FN* correspond to those incorrectly identified as mask and background, respectively. *SN* allows to quantify the proportion of voxels that belong to the ground truth mask, and can be obtained as in Equation (4) [48]:

(4) $$SN=\frac{TP}{TP+FN}$$

*OC* allows to calculate how close a finite set is from the other, in terms of overlapping [49]. A perfect overlap would have a value of 1, and two images without any overlapping should obtain 0 score. It can be calculated with Equation (5).

(5) $$OC=\frac{\sum_{i}^{N}p_{i}g_{i}}{\min\left(\sum_{i}^{N}p_{i},\sum_{i}^{N}g_{i}\;\right)}$$

*AUC* is used as a measurement of a classifier’s performance, being more complete than the usual overall accuracy [48,50], and can be obtained with Equation (6)

(6) $$AUC=1-\frac{1}{2}\left(\frac{FP}{FP+TN}+\frac{FN}{FN+TP}\right)\;$$

The measures were selected based on the guidelines proposed by Taha and Hanbury [48], attending to the following properties and requirements on 3D medical image segmentation: outliers exist (some outsider voxels might be incorrectly classified as ground truth), complex boundary (cerebellar fissures present very complex shapes and boundaries), and contour is important.

## 3. Results

This section exposes the result of evaluations performed to the three models. Table 2 allows to analyze the mean scores for the three models in the whole test set. It can be seen that the worst results were obtained by model M1. Models M2 and M3 achieved very high scores in evaluations.

For an easy understanding and analysis, we decided to divide into six subsections. The first four subsections correspond to results on each dataset used, the fifth presents our time analysis, and the last subsection corresponds to the comparison with segmentations produced by ACAPULCO and CERES.

### 3.1. Results for Our Dataset

**Figure 4:**
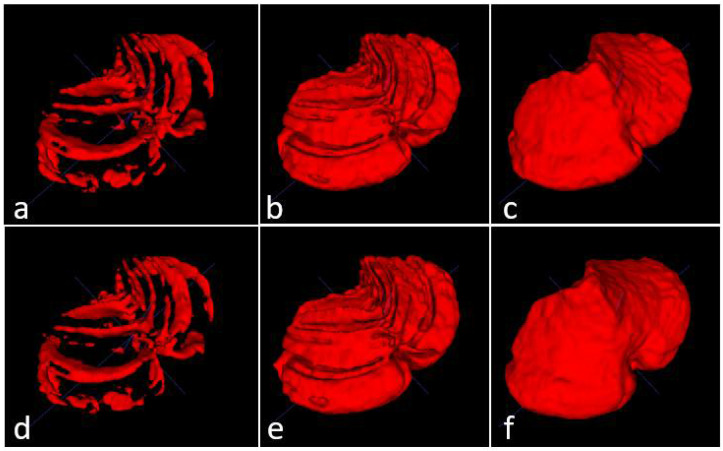
Masks and predictions for an MRI belonging to one of the SCA2 patients in the test subset. The top row shows the original masks, obtained with the procedure described in Section 2.2, and the bottom row displays the segmentations produced by our models. Cerebellar fissures in (**a**,**d**), cerebellum tissue with fissures in (**b**,**e**), and whole cerebellum without any fissure in (**c**,**f**).

The three models (see Table 1) were tested on five unseen magnetic resonance images. The test subset contained one healthy control (subject 1), two presymptomatic carriers (subjects 2 and 4), and two SCA2 patients (subjects 3 and 5). Figure 4 shows a comparison between the original masks and the segmentations produced by M1, M2 and M3. It can be appreciated the similarity between original and segmented images. Some errors remain, mainly in the contour of segmented masks; those errors will be covered in next investigations. Table 3 shows the result of the evaluations on model M1, segmenting cerebellar fissures only.

Produced segmentations have relatively good scores. Mean DSC and OC are 0.854 and 0.898, respectively. All SP are above 0.99, which means an optimal recognition of background voxels. Low SN values represent some errors in the voxels belonging to cerebellar fissures, mainly in the MRI belonging to the healthy control (0.73, the minimum SN value). It seems that the best behavior was obtained for subject 3, one of the SCA2 patients in our dataset. Note that segmenting cerebellar fissures is a difficult task and, as such, characteristics change greatly between different people. Furthermore, no postprocessing was applied to the results of model M1. Figure 4d shows an example of the outputs produced by our model, compared against the ground truth mask in Figure 4a.

Table 4 shows the evaluation results for model M2 (segmentation of cerebellum tissue with its fissures). As observed, results for this model were much better than the previous one. This is a logical result, considering that segmenting a single, larger structure, which is always located in the same place on MRI, should be easier than segmenting smaller regions with many position changes. The best scores were achieved for the subject 4 MRI, producing better segmentations. The mean values for DSC and OC are 0.973 and 0.987, respectively. SP, SN and AUC are all above 0.98, which means a good background and foreground voxel classification. Figure 4e displays an example output from this model.

Table 5 shows the results for the model segmenting the whole cerebellum (M3). As in Table 4, all scores are above 0.95, which gives the idea of a high precision in the segmentation results. Mean DSC and OC are 0.969 and 0.982, respectively. As in evaluation for model M2, SP, SN and AUC are above 0.98, which means a high-quality segmentation. In a general way, the segmentations obtained by models M1, M2 and M3 have a good quality. Models M2 and M3 obtained better scores than M1.

### 3.2. Results on Hammers Dataset

The three models were evaluated using a subset of the Hammers 2017 dataset. For this evaluation, we used the first 10 images. The images in the dataset are named from a01 to a30; we used images from a01 to a10. The images were processed with the same procedure described in Section 2.2, but manual correction of generated binary maps was not performed, as we wanted to check the possibility of automatically creating a new dataset. As a cerebellar map for the initial step, the original segmentations were conveniently corrected. Therefore, the rest of the dataset preparation was done in a fully automatic manner.

Evaluation results for model M1 on this dataset can be observed in Table A1. This time the segmentations produced were less precise. The mean DSC obtained was 0.755, while the mean overlap coefficient was 0.826. We believe that this result presents a direct relation with the fact that binary maps for each MRI were not manually corrected. A revision of those features should improve the segmentation, and it will be covered in future investigations. As in evaluation with images from our dataset, high SP and low SN and AUC were obtained, meaning that the model had some trouble identifying the tissue belonging to cerebellar fissures.

Results for model M2 are presented in Table A2. It may be observed that the scores obtained are competitive with those obtained in our dataset, as mean DSC and OC are 0.951 and 0.983, respectively. The scores in the segmentations were quite high and close to each other. Minimum DSC and OC are 0.945 and 0.975, respectively, which indicates very realistic segmentations as in previous evaluation of model M2. SP, SN and AUC are above 0.98, which demonstrates a high-quality segmentation on cerebellar tissue with fissures.

Finally, Table A3 shows the evaluations for model M3. As in Table A2, the results are very promising, giving mean DSC and OC with values of 0.947 and 0.976 respectively. The rest of calculated scores, all above 0.98, also give the notion of very good segmentations.

As in the previous case, the worst results were achieved for the model M1, in the segmentation of cerebellar fissures.

### 3.3. Results on DLBS Dataset

As a third set of MRIs for evaluating the methods, 10 images from the Dallas Lifespan Brain Study were used. For our purposes, we selected 10 MRIs belonging to older APOE-ε4 gene carriers.

Results of the evaluation on segmentations produced by model M1 can be observed in Table A4. As in the previous discussion on cerebellar fissure segmentation (Section 3.2), the DSCs are between 0.71 and 0.76. Mean DSC and OC are 0.745 and 0.799, respectively. The rest of the scores remain similar to analysis performed in our dataset and Hammers: low SN, which means errors in the precise classification of the fissures.

Evaluations for model M2 are presented in Table A5, and some improvement can be seen with respect to evaluations on Hammers dataset. Mean values of DSC and OC are 0.967 and 0.975, respectively, for a very good segmentation of cerebellum with its fissures. As expected, values of SN, SP and AUC are above 0.96.

Scores for model M3 are shown in Table A6. Again, the scores are quite good, with mean DSC and OC of 0.963 and 0.975, respectively.

### 3.4. Results on Dataset from BrainWeb

As commented in Section 2.3, seven MRIs were generated through the BrainWeb web portal, simulating multiple sclerosis. The images were created with variable parameters such as rotation, noise level, and MS severity.

Table A7 shows the scores for model M1, presenting the same situation as previous evaluations. Mean DSC and OC obtained were 0.728 and 0.81, respectively, and the sensitivity was severely affected. Table A8 shows the evaluations for model M2, with another surprising result. Achieved scores are all above 0.97, and the mean DSC and OC were 0.973 and 0.988, respectively. The same occurs with the scores for model M3 (Table A9), with 0.964 and 0.982 as mean DSC and OC, respectively. Despite the high scores achieved in this dataset, we believe that further analysis should be performed, as all images are created from two original templates: one for severe MS, and one for mild and moderate MS.

### 3.5. Time Analysis

An analysis was performed to evaluate the time our architecture takes to segment new images. All experiments were carried out on a Lenovo computer, equipped with an Intel Core i3-8145U processor, and 8 GB RAM. Table 6 shows the mean times for models M1, M2 and M3, as well as preprocessing and load times.

The load times for each dataset are small, ranging from 0.04 to 0.07 s. Preprocessing times ranged from 177.95 to 265.75 s. This is the most time-consumer phase in our pipeline, as it involves bias field correction, image registration, normalization, histogram equalization, and cropping.

For model M1, the best segmentation times were obtained over Hammers subset, with a mean processing time of 49.89 s per image. The global mean time of this model was 53.43 s. Segmentation times for M2 were slightly higher, averaging 54.55 s. The best results were also obtained for Hammers subset, with a mean time of 50.21 s. Finally, results of time analysis for model M3 were better on Hammers subset, with a mean of 50.42 s. The mean time for all the images was 55.23 s.

In a general manner, the total time needed for processing an MRI is the sum of loading, preprocessing and segmentation tasks. Since our three models work with the same cropped portion of the preprocessed MRI, the load and preprocessing operations are performed only once on each image. The total time for every image is then the sum of loading, preprocessing, and segmentations for M1, M2 and M3. The total mean time of processing for our models was 385.26 s (about six minutes for each image). Considering that manual segmentation can take several hours for each MRI, we believe that it is a remarkable advance in such task. However, finding a faster BFC/registration technique should greatly improve this result, as preprocessing is the most time-consuming phase of our process.

### 3.6. Comparison with Other Methods

For stablishing an improvement on cerebellar tissue segmentation with special attention to fissures, comparisons were made with ACAPULCO and CERES. We compared the results of our model M2 with the segmentations produced by these two tools. Segmentations from ACAPULCO were obtained using the docker image that the authors made available in the original paper [1], and segmentations from CERES were obtained through a web portal available to the public, also shared by the authors on their paper [6].

As these are tools for cerebellar parcellation, a binary mask of the whole cerebellum was obtained for each segmentation, constructed by combining all the labels in the segmented images. The evaluations were performed on the five test magnetic resonances of our cohort, and the 10 images from the DLBS dataset. The measures used for the comparison were dice score (DSC), overlap coefficient (OC), and specificity (SP). Table 7 shows the comparison of DSC in our images.

As can be seen, our model M2 achieved higher DSC than both methods. Mean DSC were 0.973, 0.903 and 0.924 for M2, Acapulco and CERES, respectively. CERES performed better than ACAPULCO in the segmentation, but in general both methods only identify the largest fissures, and a substantial part of the small fissures is misclassified. We think that this event is related with the segmentations used in both methods as a training/knowledge base, since both methods were used without any modification. The best behavior for both methods was on segmenting the first resonance image, corresponding to a healthy control.

**Figure 5:**
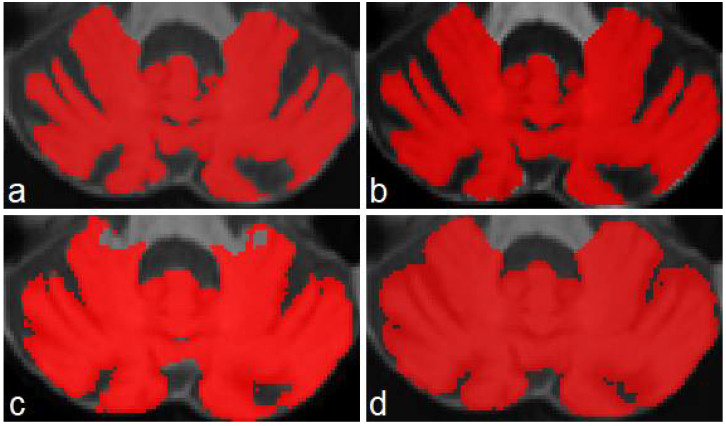
Example of segmentations produced by the approaches for a sample image from our dataset. Original mask (**a**), followed by segmentation produced by M2 (**b**), CERES (**c**) and ACAPULCO (**d**).

Figure 5 presents an example of segmentation produced by the three models for a subject in our dataset. As the figure shows, ACAPULCO (Figure 5d) only detected parts of the biggest fissures, while the smaller ones are classified as cerebellar tissue. CERES (Figure 5c) recognized fissures better than ACAPULCO, but some of them are also misclassified. Furthermore, some irregularities are present in the front of the cerebellum, leaving some holes in the mask produced by CERES. Segmentations obtained by model M2 (Figure 5b) are very close to the real ones, correctly recognizing most of the fissures.

Table 8 shows a comparison for the OC scores achieved by the three methods. Mean scores for M2, ACAPULCO and CERES were 0.987, 0.994 and 0.988, respectively. Results are very close between approaches, but in general terms, ACAPULCO achieved higher OC scores. This is a logical conclusion, as ACAPULCO tends to misclassify fissures. As a result, the original masks are almost entirely contained in segmentations produced by ACAPULCO. The same happens with segmentations produced by CERES.

In Table 9 are included the results of the SP analysis for the three models. It can be appreciated that M2 model achieved the higher scores, followed by CERES, and finally ACAPULCO. The mean values are 0.994, 0.971 and 0.964, respectively.

Table 7, Table 8 and Table 9 clearly indicate that model M2 produced better segmentations than ACAPULCO and CERES. Higher DSC and SP combined with lower OC, means that our approach correctly identifies the most of fissures on the cerebellum.

Table 10 shows the DSC comparison for the DLBS subset. The three approaches obtained close dice scores, with a mean value of 0.967, 0.931 and 0.945, respectively. The 10 images for this comparison belong to healthy controls, which means less fissures, so the scores for ACAPULCO and CERES were increased.

**Figure 6:**
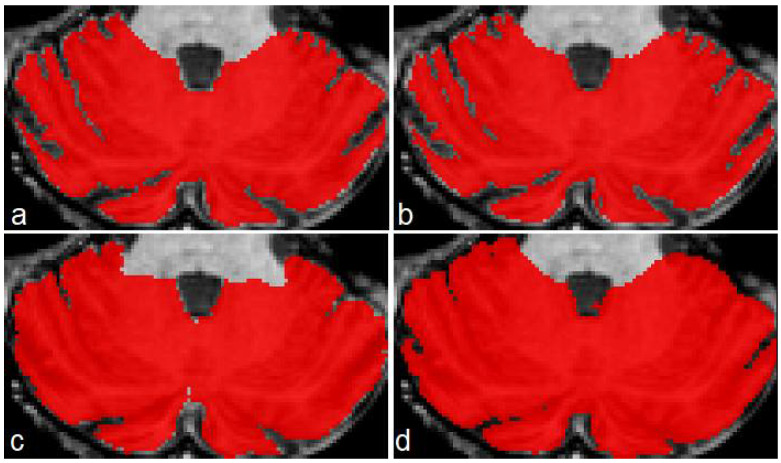
Segmentations produced by the three approaches for a sample image from the DLBS dataset. Original mask (**a**), followed by segmentation produced by M2 (**b**), CERES (**c**) and ACAPULCO (**d**).

Figure 6 shows a case of the segmentations produced for this second dataset. As in the previous example, the best segmentations were produced by model M2 (Figure 6b). There are some irregularities on borders, which we think can be corrected by applying some postprocessing technique (rather than longest connected component, which is the only postprocessing we currently apply on segmentations). In this example, ACAPULCO was capable of segmenting some fissures better than CERES (Figure 6c,d).

Table 11 shows a comparison for the OC scores achieved in the DLBS dataset. Higher values were obtained by ACAPULCO, followed by CERES, and finally M2. The mean values were 0.998, 0.980 and 0.975, respectively. This represents the same phenomena as Table 8: segmentations produced by ACAPULCO and CERES include the original masks because of the problems when recognizing cerebellar fissures, resulting in elevated OC.

In Table 12 are included the SP scores achieved in the DLBS dataset. As in Table 9, model M2 presented the best behavior, which means that the classification of background voxels was better. Mean scores were 0.993, 0.970 and 0.977, respectively.

Results for this dataset were similar to those obtained in our five test MRIs. The model M2 presented higher DSC and SP, and lower OC than ACAPULCO and CERES. This means that M2 identifies cerebellar fissures better than the other approaches.

## 4. Discussion

Three models have been proposed for segmentation tasks on human cerebellum from magnetic resonance imaging: the first model (M1) segments cerebellar fissures, the second (M2) segments the cerebellum with the most of its fissures, and the third (M3) obtains the whole cerebellum without fissures. The three models were tested on a total of 32 MRIs, composed of 21 healthy controls, four SCA2 patients, and seven MRIs with multiple sclerosis.

In the case of cerebellar fissure segmentation (model M1), the best DSC obtained was 0.895 in our dataset, and the worst case presented a score of 0.707 in the Hammers dataset. We observed that the best results were achieved on the MRIs of SCA2 patients with severe atrophy, indicating that the model might not be capable of correctly find the fissures in healthy people. More tests need to be done to verify if the proposed U-Net architecture can be modified in any way, or more augmentation techniques/training epochs are necessary for improving segmentation results. A postprocessing stage could be added too, increasing the possibility of producing better segmentations. Despite the low results (minimum DSC = 0.707), we have not seen other investigations dedicated to specifically segmenting and quantifying the cerebellar fissures, and we consider this to be a good starting point for future researches on this kind of study.

The model for segmentation of the cerebellum with its fissures (M2) presented very precise results, with DSC ranging from 0.946 to 0.981 among the four subsets used for testing. This result implies that volumetric calculations might be performed in the human cerebellum, with a higher grade of precision. We think that the model could be integrated in some greater pipeline for characterizing neurodegenerative diseases. The model performed well on MRIs of healthy people and patients, making it suitable for the task.

The model for segmenting the whole cerebellum (M3) also obtained very good results, with dice scores ranging from 0.946 to 0.980, demonstrating very precise segmentations in the 32 test images. Obtained scores highly reduce the chance of overfitting during training process, and allow the affirmation that models have sufficient generalization for working with images from different origins.

Segmentations produced by the models M2 and M3 could be used to improve current cerebellar segmentation/parcellation methods, obtaining more accurate volumetric estimations on patients with cerebellar degeneration caused by SCAs or other neurodegenerative diseases. Furthermore, the procedure proposed in Section 2.2 for the creation of our dataset can be adapted to any research with the same interests, always providing the correct mask at the beginning.

The three models present good performance in terms of efficiency, as total time needed when processing a new image is about six minutes (less than three minutes if the loading and preprocessing stages are not considered).

The model M2 was compared with two state-of-the-art approaches, obtaining better scores in all cases. The comparison was only made with 15 resonance images, and deeper comparisons will be performed in future researches.

Based on analysis results, we may conclude that convolutional neural networks can be applied on segmenting complicated features from brain magnetic resonances. Not only well-defined organs such as cerebellum, but also fissures can be obtained, always providing the correct dataset and adequate training. Our model trained for cerebellar fissures did not obtain such high scores as expected, but we think that fissures can be obtained by combining outputs of models M2 and M3.

The outcomes of this study should provide a comprehensive set of tools to specialists in neurodegenerative diseases. Digital tools can be generated and incorporated into existing visualization applications, increasing the speed and precision in diagnosis and characterization.

For more in-depth evaluation of the proposed method, larger datasets must be tried, as well as other CNN architectures, with different grades of complexity, and a higher number of features. In future research we aim to integrate the models described here into more complex architectures and pipelines, such as cerebellum parcellation.

## 5. Conclusions

This article has evaluated the possibility of applying convolutional neural networks for automatically segmenting the cerebellum and its fissures from brain magnetic resonance imaging. Three models, built upon the same U-Net based architecture, have been proposed for segmenting cerebellar fissures, cerebellum with all fissures, and cerebellum without any fissures. Analysis has been performed on 32 MRIs, including healthy controls, presymptomatic carriers, SCA2 patients, and multiple sclerosis patients. The best dice scores achieved were 0.895, 0.981 and 0.98 on each task, respectively. The proposed architecture is highly efficient, since segmentations can be carried on in less than a minute after preprocessing. Analysis results indicate that convnets are capable of segmenting the human cerebellum with high precision. The model prepared for segmenting the cerebellum with its fissures was compared with two existent methods, achieving better results than both in all tests. The images resulting from the segmentations could be incorporated into higher pipelines, dedicated to diagnosing or characterizing any disease that affects the cerebellum, and could help to improve the estimation of volume loss and general damage to the cerebellum. Furthermore, a simple method has been proposed for facilitating the construction of similar datasets. The use of the procedure should help to quickly construct datasets, saving time and efforts.

## Figures and Tables

**Table 2:** Mean scores for models M1, M2 and M3 in the whole set of test images.

	M1	M2	M3
DSC	0.761	0.965	0.959
OC	0.826	0.982	0.978
SP	0.997	0.992	0.991
SN	0.749	0.977	0.969
AUC	0.871	0.985	0.980

**Table 3:** Evaluation results for model M1 in our dataset.

	Subject 1	Subject 2	Subject 3	Subject 4	Subject 5
DSC	0.803	0.895	0.864	0.834	0.875
OC	0.882	0.914	0.924	0.895	0.876
SP	0.998	0.998	0.997	0.999	0.996
SN	0.737	0.877	0.924	0.780	0.875
AUC	0.868	0.938	0.960	0.889	0.935

**Table 4:** Evaluation results for cerebellar tissue with fissures (model M2) in our dataset.

	Subject 1	Subject 2	Subject 3	Subject 4	Subject 5
DSC	0.976	0.977	0.970	0.981	0.965
OC	0.991	0.984	0.992	0.992	0.977
SP	0.993	0.995	0.995	0.995	0.994
SN	0.991	0.984	0.992	0.992	0.977
AUC	0.992	0.989	0.993	0.994	0.986

**Table 5:** Evaluation results for whole cerebellum segmentation without fissures (model M3).

	Subject 1	Subject 2	Subject 3	Subject 4	Subject 5
DSC	0.976	0.975	0.954	0.980	0.963
OC	0.984	0.980	0.987	0.986	0.976
SP	0.994	0.994	0.991	0.995	0.991
SN	0.984	0.980	0.987	0.986	0.976
AUC	0.989	0.987	0.989	0.991	0.984

**Table 6:** Mean times for loading, preprocessing, and segmentation processes. From left to right column are presented: dataset names, load times, preprocessing times, and segmentation time for M1, M2 and M3. The time is expressed in seconds (s).

	Load	Preprocessing	Segmentation		
			**M1**	**M2**	**M3**
Ours	0.06	227.57	53.40	51.44	53.00
Hammers	0.06	263.77	49.89	50.24	50.42
DLBS	0.07	206.73	55.85	60.26	56.90
Brainweb	0.04	180.06	55.08	54.75	61.34

**Table 7:** DSC comparison between our M2 model, ACAPULCO and CERES.

	S.1	S.2	S.3	S.4	S.5
M2	0.976	0.977	0.970	0.981	0.965
ACAPULCO	0.910	0.905	0.894	0.909	0.900
CERES	0.935	0.927	0.911	0.924	0.926

**Table 8:** OC comparison between our M2 model, ACAPULCO and CERES.

	S.1	S.2	S.3	S.4	S.5
M2	0.991	0.984	0.992	0.992	0.977
ACAPULCO	0.999	0.990	0.990	0.996	0.999
CERES	0.991	0.989	0.981	0.991	0.988

**Table 9:** SP comparison between our M2 model, ACAPULCO and CERES.

	S.1	S.2	S.3	S.4	S.5
M2	0.993	0.995	0.995	0.995	0.994
ACAPULCO	0.965	0.967	0.966	0.971	0.953
CERES	0.973	0.973	0.980	0.977	0.955

**Table 10:** DSC comparison between the approaches, in DLBS subset.

Subject No.	M2	ACAPULCO	CERES
1	0.969	0.926	0.945
2	0.965	0.939	0.950
3	0.960	0.938	0.941
4	0.966	0.936	0.950
5	0.978	0.922	0.953
6	0.962	0.930	0.946
7	0.971	0.937	0.954
8	0.960	0.911	0.921
9	0.967	0.935	0.944
10	0.974	0.936	0.954

**Table 11:** OC comparison between the approaches, in DLBS subset.

Subject No.	M2	ACAPULCO	CERES
1	0.970	0.996	0.983
2	0.974	0.999	0.975
3	0.971	0.993	0.982
4	0.969	0.998	0.984
5	0.987	0.995	0.982
6	0.981	0.994	0.975
7	0.977	0.998	0.981
8	0.964	0.999	0.980
9	0.975	0.998	0.985
10	0.982	0.995	0.987

**Table 12:** SP comparison between the approaches, in DLBS subset.

Subject No.	M2	ACAPULCO	CERES
1	0.995	0.969	0.978
2	0.992	0.970	0.979
3	0.991	0.972	0.971
4	0.994	0.973	0.975
5	0.995	0.972	0.976
6	0.992	0.973	0.982
7	0.996	0.969	0.983
8	0.993	0.973	0.982
9	0.993	0.970	0.980
10	0.995	0.961	0.968

## Data Availability

At the time of writing this paper, the original images are being uploaded to https://github.com/robbinc91/cerebellar_fissures_segmentation_cnn (last accessed on 8 December 2021). The computer codes for creating the dataset are also being shared, allowing other researchers to replicate our study. The rest of datasets used in this research are publicly available on the internet. The hammers 2017 dataset can be accessed from http://brain-development.org/ (last accessed on 5 December 2021), simulated multiple sclerosis images can be accessed from http://www.bic.mni.mcgill.ca/brainweb/ (last accessed on 5 December 2021), and DLBS dataset may be obtained from https://fcon_1000.projects.nitrc.org/indi/retro/dlbs.html (last accessed on 5 December 2021).

## Appendix A

This appendix contains the tables for the evaluation results on the 10 first images from Hammers, DLBS and BrainWeb datasets.

**Table A1:** Evaluation results for cerebellar fissures (model M1) on Hammers subset.

Subject No.	DSC	OC	SP	SN	AUC
1	0.740	0.908	0.999	0.624	0.812
2	0.752	0.864	0.998	0.665	0.832
3	0.816	0.828	0.998	0.805	0.901
4	0.789	0.828	0.998	0.753	0.875
5	0.726	0.729	0.996	0.722	0.859
6	0.838	0.855	0.997	0.855	0.926
7	0.724	0.773	0.998	0.773	0.886
8	0.735	0.799	0.996	0.799	0.897
9	0.732	0.847	0.998	0.645	0.821
10	0.707	0.833	0.998	0.614	0.806

**Table A2:** Evaluation results for cerebellar tissue with fissures (model M2) on Hammers subset.

Subject No.	DSC	OC	SP	SN	AUC
1	0.945	0.986	0.988	0.986	0.987
2	0.966	0.976	0.993	0.976	0.985
3	0.945	0.990	0.986	0.990	0.988
4	0.954	0.982	0.988	0.982	0.985
5	0.952	0.981	0.987	0.981	0.984
6	0.947	0.989	0.984	0.989	0.987
7	0.949	0.992	0.986	0.992	0.989
8	0.953	0.975	0.986	0.975	0.980
9	0.951	0.988	0.988	0.988	0.988
10	0.950	0.976	0.987	0.976	0.982

**Table A3:** Evaluation results for whole cerebellum segmentation, without fissures (model M3) on Hammers subset.

Subject No.	DSC	OC	SP	SN	AUC
1	0.950	0.973	0.990	0.973	0.981
2	0.955	0.958	0.992	0.958	0.975
3	0.938	0.985	0.983	0.985	0.984
4	0.949	0.978	0.986	0.978	0.982
5	0.944	0.972	0.984	0.972	0.978
6	0.945	0.982	0.983	0.982	0.982
7	0.943	0.992	0.984	0.992	0.988
8	0.953	0.977	0.985	0.977	0.981
9	0.950	0.979	0.988	0.979	0.983
10	0.946	0.965	0.987	0.965	0.976

**Table A4:** Evaluation results for cerebellar fissures (model M1) on DLBS dataset.

Subject No.	DSC	OC	SP	SN	AUC
1	0.776	0.813	0.997	0.813	0.905
2	0.738	0.833	0.997	0.833	0.915
3	0.730	0.858	0.997	0.858	0.927
4	0.723	0.724	0.997	0.723	0.860
5	0.720	0.794	0.998	0.658	0.828
6	0.769	0.776	0.998	0.763	0.880
7	0.738	0.752	0.997	0.724	0.861
8	0.723	0.875	0.998	0.616	0.807
9	0.719	0.754	0.997	0.664	0.875
10	0.769	0.820	0.999	0.725	0.862

**Table A5:** Evaluation results for cerebellar tissue with fissures (model M2) on DLBS dataset.

Subject No.	DSC	OC	SP	SN	AUC
1	0.969	0.970	0.995	0.970	0.983
2	0.965	0.974	0.992	0.974	0.983
3	0.960	0.971	0.991	0.971	0.981
4	0.966	0.969	0.994	0.969	0.982
5	0.978	0.987	0.995	0.987	0.991
6	0.962	0.981	0.992	0.981	0.987
7	0.971	0.977	0.996	0.966	0.981
8	0.960	0.964	0.993	0.964	0.978
9	0.967	0.975	0.993	0.975	0.984
10	0.974	0.982	0.995	0.982	0.989

**Table A6:** Evaluation results for whole cerebellum segmentation, without fissures (model M3) on DLBS dataset.

Subject No.	DSC	OC	SP	SN	AUC
1	0.968	0.978	0.993	0.978	0.985
2	0.965	0.980	0.991	0.980	0.985
3	0.964	0.987	0.990	0.987	0.988
4	0.963	0.989	0.993	0.969	0.981
5	0.963	0.963	0.994	0.963	0.979
6	0.959	0.978	0.990	0.978	0.984
7	0.969	0.969	0.994	0.969	0.981
8	0.948	0.961	0.993	0.935	0.964
9	0.964	0.973	0.991	0.973	0.982
10	0.968	0.972	0.995	0.972	0.983

**Table A7:** Evaluation results for cerebellar fissures (model M1) on BrainWeb dataset.

Subject No.	DSC	OC	SP	SN	AUC
1	0.722	0.729	0.993	0.714	0.854
2	0.711	0.932	0.993	0.691	0.842
3	0.715	0.724	0.993	0.706	0.849
4	0.722	0.938	0.993	0.707	0.850
5	0.739	0.781	0.994	0.701	0.847
6	0.754	0.801	0.994	0.713	0.853
7	0.738	0.767	0.993	0.912	0.852

**Table A8:** Evaluation results for cerebellar tissue with fissures (model M2) on BrainWeb dataset.

Subject No.	DSC	OC	SP	SN	AUC
1	0.973	0.986	0.996	0.961	0.979
2	0.972	0.981	0.995	0.964	0.979
3	0.973	0.985	0.996	0.962	0.979
4	0.973	0.984	0.996	0.962	0.979
5	0.974	0.980	0.995	0.969	0.982
6	0.974	0.989	0.995	0.970	0.982
7	0.974	0.982	0.995	0.966	0.981

**Table A9:** Evaluation results for whole cerebellum segmentation, without fissures (model M3) on BrainWeb dataset.

Subject No.	DSC	OC	SP	SN	AUC
1	0.965	0.983	0.995	0.949	0.972
2	0.964	0.982	0.995	0.946	0.970
3	0.965	0.982	0.995	0.948	0.971
4	0.965	0.983	0.995	0.948	0.971
5	0.963	0.982	0.995	0.944	0.969
6	0.963	0.983	0.995	0.944	0.970
7	0.963	0.981	0.995	0.945	0.970
